# Estimation of the Farm-Level Basic Reproduction Number for African Swine Fever Outbreaks in the Philippines

**DOI:** 10.1155/tbed/6946683

**Published:** 2025-10-23

**Authors:** Chia-Hui Hsu, Rachel Schambow, Maximino Montenegro, Ruth Miclat-Sonaco, Andres Perez

**Affiliations:** ^1^Center for Animal Health and Food Safety, College of Veterinary Medicine, University of Minnesota, Saint Paul 55108, Minnesota, USA; ^2^Pig Improvement Company (PIC) Philippines, Pasig 1605, Philippines; ^3^National Livestock Program, Office of the Undersecretary for Livestock, Department of Agriculture, Elliptical Road, Diliman, Quezon City 1101, Philippines

**Keywords:** African swine fever, basic reproduction number, disease dynamics, endemicity, philippine

## Abstract

African swine fever (ASF) genotype II has severely impacted the Philippine swine industry since 2019, affecting backyard and commercial farms as well as native wild boar populations, with significant economic and food security implications. Understanding ASF transmission dynamics is crucial for effective control and management strategies. However, farm-level transmission patterns remain unclear, and a comprehensive analysis incorporating both national and island-specific estimates has yet to be conducted. Using ASF outbreak data from 2019 to 2022, we estimated the herd-level basic reproduction number (*R*_*h*_) for the Philippines and its three major islands—Luzon, Visayas, and Mindanao—using deterministic and stochastic models. Epidemic descriptive analysis and Spearman's correlation results indicate that Luzon's data most accurately reflect national epidemic trends. Our estimation model for *R*_*h*_ shows that while the long-term trend of ASF approaches a threshold near 1, indicating a shift toward endemicity, the *R*_*h*_ value does not consistently remain below 1 during the study period, suggesting ongoing outbreaks. Luzon, the outbreak's origin with the highest swine farm density, mirrors national trends, whereas Mindanao and Visayas exhibit distinct transmission patterns influenced by local production systems and swine demographics. These results highlight the need for region-specific ASF management strategies, including enhanced biosecurity in Luzon and community-based surveillance in Visayas and Mindanao. Adapting control measures to regional transmission patterns can improve ASF management and support the long-term recovery of the Philippine swine industry.

## 1. Introduction

African swine fever (ASF) is a severe hemorrhagic viral disease of rising global concern. It is caused by ASF virus (ASFV), a double-stranded DNA arbovirus originally identified in Africa [1]. ASFV infects members of the family Suidae, which includes domestic pigs, wild boars, bushpigs, and other related wild species. Although ASFV is not zoonotic to humans, affected domestic pig populations experience a significant mortality rate, reaching nearly 100% with highly virulent strains. The disease has far-reaching implications for pig farming, wildlife conservation, and the global economy. Due to its substantial impact on the swine industry, ASF is classified as a notifiable disease by the World Organization for Animal Health (WOAH) [2].

Since 2007, the highly virulent genotype II strain, known as Georgia 07, emerged in the country of Georgia [3, 4] and rapidly spread to neighboring regions. This strain has since caused an ASF pandemic across Europe, Asia, the Dominican Republic and Haiti, and the Asia Pacific region [5–7]. ASFV reached the Philippines in July 2019 [8, 9], where it has affected both backyard and commercial farms as well as wild pig populations across many regions [10]. The widespread presence of ASF across the Philippines has a ripple effect, threatening the swine supply chain, food security, economic stability, and biodiversity. Between 2019 and 2024, ASF has continued to pose significant challenges to the swine industry in the Philippines, despite the government's implementation of various control and management programs [10].

The main initiatives to combat ASF include the Bantay ASF sa Barangay Program, the National ASF Prevention and Control Program (NASFPCP), the Integrated National Swine Production Initiatives for Recovery and Expansion (INSPIRE) Program, and the National Task Force on Animal-Borne Diseases (NTFAD). These programs are led by the Department of Agriculture and the Bureau of Animal Industry [10, 11], and they emphasize proactive measures to prevent and control the disease. Spatial epidemiology research indicates that ASF exhibited recurrent patterns in the Philippines from 2019 to 2022 [12], with various factors hindering eradication efforts. Understanding and quantifying the transmission dynamics of ASF is crucial for assessing control measures and informing future government interventions for more effective disease management.

The basic reproduction number (*R*_0_) is a key epidemiological metric that quantifies how a disease spreads within an animal population [13]. It represents the average number of secondary infections generated by one single infected individual in a completely susceptible population. If the value of *R*_0_ > 1, the disease is expected to spread and result in an epidemic; if *R*_0_ = 1, the disease becomes endemic in the study area; and if *R*_0_ < 1, it suggests the disease is approaching eradication. On an individual animal level, *R*_0_ provides insights into how contagious a disease is, and it can be further used to estimate vaccine coverage required to achieve herd immunity. Here, we extend the concept of *R*_0_ to the herd level (*R*_*h*_), and in the context of ASF outbreaks in the Philippines, we estimated the average number of secondary farm outbreaks caused by one single ASF-infected farm. The primary objective of this study was to estimate the reproduction number of ASF outbreaks at farm level in the Philippines from 2019 to 2022, using national-level data as well as data stratified by island (Luzon, Visayas, and Mindanao), to compare transmission dynamics and identify distinct patterns across regions. This comprehensive approach assesses the dynamics of ASF in the Philippines from 2019 to 2022 and supports the development of targeted control strategies.

## 2. Materials and Methods

### 2.1. Data

Officially reported farm-level ASF outbreak data were obtained from the International Training Center on Pig Husbandry (ITCPH), Agricultural Training Institute, Department of Agriculture, Philippines and the Bureau of Animal Industry. The dataset covered a 36-month period from August 16, 2019 to July 20, 2022. The national outbreak data were categorized into three major island groups: Luzon, Visayas, and Mindanao, enabling region-specific analyses. Geographic coordinates of outbreak locations were represented using the centroids of the latitude and longitude of barangays, the smallest administrative units in the Philippines, facilitating accurate spatial analysis. For each farm-level outbreak, the dataset included a unique event ID, hierarchical administrative information (region, province, municipality, and barangay), and the date of reporting. Outbreak data were organized by week (ISO week date system) and stored in a standardized database. Descriptive statistics were calculated using Microsoft Excel (version 2016) and R software (version 4.2.2).

### 2.2. Descriptive Disease Dynamics

Epidemic curves were plotted to illustrate the temporal progression of the ASF outbreak at the national level and for each island level individually. The analysis included weekly cumulative case counts, total outbreak weeks, and a 3-week moving average (MA) adjustment to smooth fluctuations and highlight trends. This approach provided understanding of the weekly outbreak scale and the severity of reported cases over time.

### 2.3. Kernel Density Estimation (KDE)

KDE is a nonparametric statistical method used to estimate the probability density function, which is frequently applied in epidemic outbreak analysis [14]. KDE was used to visualize ASF outbreak hotspots in Luzon, Visayas, and Mindanao islands. This method mapped the density of positive cases using geographic coordinates (barangay centroids) to identify high-risk areas at the city and municipality levels, assisting local veterinary services in pinpointing outbreak clusters. KDE plots highlighted areas with higher outbreak intensity, represented by a color gradient from green to red (red represent high index). The analysis was conducted using ArcGIS Pro (version 3.1.0, ESRI Inc., Redlands, CA, USA).

### 2.4. Spearman Correlation

Spearman's rank correlation (*ρ*) was used to measure how closely the ASF outbreak trends on each island (Luzon, Visayas, and Mindanao) aligned with the nationwide outbreak trend.

(1) $$\begin{array}{c} \rho =1-\frac{6\sum d_{i}^{2}}{n\left(n^{2}-1\right)}. \end{array}$$

The correlation coefficient quantified the strength and direction of the relationship between the ranked weekly case counts. Pairwise comparisons were calculated, and the analysis was conducted using the stats package in R software (version 4.2.2), and represented how well each island's outbreak dynamics matched the national trend.

### 2.5. Reproduction Ratio (*R*_*h*_) Estimation

In this study, the reproduction ratio at herd level (*R*_*h*_) was estimated at both national and island levels using farm outbreak data to model disease dynamics and the average number of secondary infections caused by one ASF-infected farm. An *R*_*h*_ greater than 1 indicates that the outbreak will continue to spread to a broader geographic area or population, as each infection generates more than one new secondary outbreak. An *R*_*h*_ less than 1 suggests the outbreak will eventually decline, while an *R*_*h*_ equal to 1 implies a stable outbreak size, potentially leading to endemicity. Estimates were calculated for the entire country and separately for Luzon, Visayas, and Mindanao, using data from ASF outbreaks with officially complete reporting dates. The *R*_*h*_ values were derived using the doubling time method, which is appropriate for scenarios where detailed swine population data are unavailable [15, 16].

(2) $$\begin{array}{c} R_{h}=1+D\;\times \ln\frac{\frac{C_{t}}{C_{0}}}{t}\;, \end{array}$$

where *D* represents the duration of the infectious period (in days), *C*_*t*_ is the number of positive reported cases at time *t*, and *C*_0_ is the number of cases at the beginning of the study (for the national level or island level, at the time of first introduction case). By defining *t* as *T*_*d*_ (the time required for cases to double), where *C*_*t*_ = 2,

Equation (2) could be simplified as follows:

(3) $$\begin{array}{c} R_{h}=1+\frac{D}{T_{d}}\ln2. \end{array}$$

*R*_*h*_ was estimated using the doubling interval method to analyze farm outbreaks, beginning with the first reported outbreak on each island and nationwide. Since the infectious period at the farm level can differ significantly from that at the individual pig level—varying widely due to factors such as farm type (backyard or commercial farms), farm size, detection time, and depopulation—*R*_*h*_ was calculated across a range of infectious period values specific to farms. To address uncertainty, both a deterministic model and a stochastic model were employed for the analysis. The deterministic model offers a simple and transparent approach to estimating disease transmission; however, it does not capture the complexity associated with factors such as the latent period, delays in detection and reporting, and the natural history of the disease. By integrating both deterministic and stochastic methods, the analysis provides a more comprehensive understanding of transmission dynamics.

#### 2.5.1. Deterministic Model

Based on Terrestrial Animal Health Code from the WOAH reference at individual animal level, the ASF incubation period ranges from 4 to 19 days, while the infectious period varies within a defined range [17–19]. In this deterministic model, we calculated the reproduction number (*R*_*h*_) across the full range of infectious periods (Supporting Information 1). To simplify the presentation and ensure clear comparisons, we observed that patterns and relative values were consistent across different infectious period scenarios. Therefore, we selected the minimum infectious period of 6 days as a representative case [17], effectively capturing the outbreak dynamics across islands while maintaining clarity and focus in the analysis.

#### 2.5.2. Stochastic Model

Estimating infectious duration at the farm level is more complex than for individual swine. Therefore, a stochastic approach was used to account for variability and uncertainty in model parameters. In the doubling time *R*_*h*_ method, the latent period (D latent, Gamma [19.9, 0.39; mean 7.8 days]), detection and reporting time (D detect, Pert distribution; min = 9, most likely = 25, and max = 32 days), and depopulation time (D depop) were based on previously estimated herd-level transmission parameters relevant to this study's scenario [20, 21]. The stochastic model of infectious period of ASF was implemented in R (version 4.2.2) using the mcd2 package with 10,000 Markov chain Monte Carlo (MCMC) simulations. The corresponding *R*_*h*_ parameters were estimated and presented with 95% confidence intervals using boxplots.

#### 2.5.3. Time-Dependent Reproductive (TD-R) Numbers

To evaluate the robustness of the results to the analytical method used to compute *R*_*h*_, an alternative algorithm was used to estimate *R*_*h*_. The effective TD-R numbers for ASF were estimated using a likelihood-based approach implemented with the R0 package in R (version 4.2.2). TD-R was calculated by averaging across all transmission networks consistent with the observed cases. Weekly incidence data were aggregated to minimize the occurrence of zero values in the time series. For weeks with no reported cases, the number of new cases was set to 1, based on the reasonable assumption that at least one farm outbreak may have been missed or underreported, a likely scenario in the Philippines. Additionally, the generation time distribution that best fit the observed case occurrence was estimated. The number of secondary cases for each primary case was then determined by averaging across all possible transmission chains derived from the epidemic curve.

#### 2.5.4. Sensitivity Analysis

To evaluate the potential impact of under-reporting on *R*_*h*_, we conducted a scenario-based sensitivity analysis. Outbreak trajectories were simulated under a range of plausible reporting rates, assuming that official reports in the Philippines captured between 50% and 90% of true outbreaks. This corresponded to adjustment factors of 1.11–2.00 applied to the observed case counts. For each adjustment factor, 500 stochastic simulations were generated in R (packages dplyr and mc2d), and the resulting epidemic curves were compared with the observed epidemic data.

### 2.6. Auto-Regressive Integrated MA (ARIMA) Model

An ARIMA model was applied to investigate temporal patterns and potential seasonality in ASF outbreaks at the national and island levels. The ARIMA model was used to analyze trends, identify repetitive seasonal effects, and forecast future outbreak dynamics based on historical data within the dataset. Model selection involved determining the optimal orders of autoregressive (AR), differencing (I), and MA components by assessing the stationarity of the time series and its autocorrelation structure.

The autocorrelation function (ACF) and partial ACF (PACF) plots were utilized to guide model parameter selection. The ACF identified correlations between observations at various lags, while the PACF determined the order of the AR terms by isolating the direct correlations at specific lags. This structured approach ensured that the ARIMA model accurately captured the temporal dynamics of ASF outbreaks in the Philippines.

To account for the declining trend in outbreaks, the ASF outbreak data were normalized on a weekly basis. Using the first outbreak week on each island as the starting point, a 52-week cycle was defined. For each week (*W*_i_) within a given year, the proportion of outbreaks was calculated relative to the total number of outbreaks for that year, adjusting for overall prevalence. The normalized data were then subjected to ACF and PACF analyses to discover temporal dependencies and cyclical patterns while mitigating the influence of decreasing prevalence. All analyses were conducted in R using the forecast and tseries packages [22].

## 3. Results

### 3.1. Descriptive Analysis

**Figure 1:**
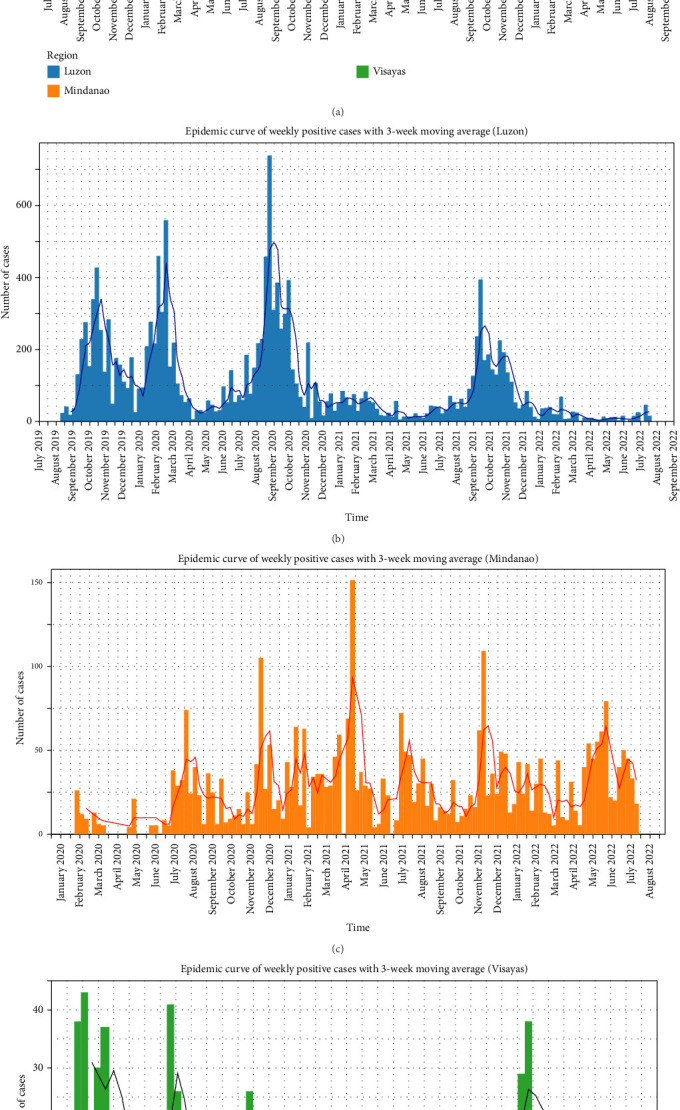
Epidemic curve of weekly reported positive cases with a 3-week moving average. (a) National, (b) Luzon, (c) Mindanao, and (d) Visayas.

An ASF epidemic curve was generated on a weekly basis using a 3-week MA, plotted separately for the national level and each of the main islands: Luzon, Mindanao, and Visayas. While the timelines for Luzon and the national data align, Mindanao reported its first ASF cases in January 2020, and Visayas observed its initial cases in January 2021 (Figure 1). Although no clear trend was detected in Mindanao, Visayas showed a relatively high number of cases in April 2021 and April 2022.

**Figure 2:**
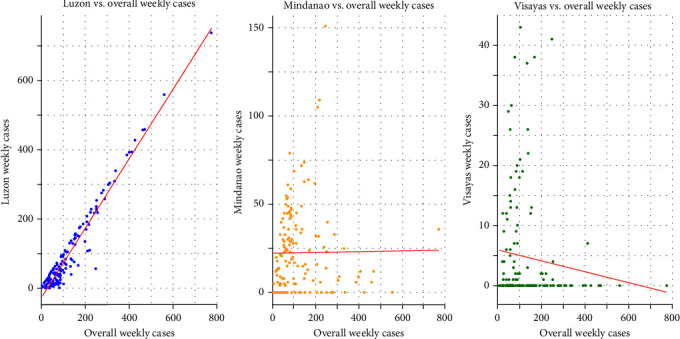
Spearman correlation. This figure shows the Spearman correlation between weekly ASF cases in Luzon, Mindanao, and Visayas against overall national weekly cases in the Philippines. Luzon exhibits a strong positive correlation (rho = 0.8836, *p* < 0.001), reflecting its dominant contribution (79%) to the total cases. Mindanao shows a weak positive correlation (rho = 0.0951, *p* = 0.241), while Visayas has a weak negative correlation (rho = −0.1337, *p* = 0.098), indicating limited associations with the overall cases.

**Figure 3:**
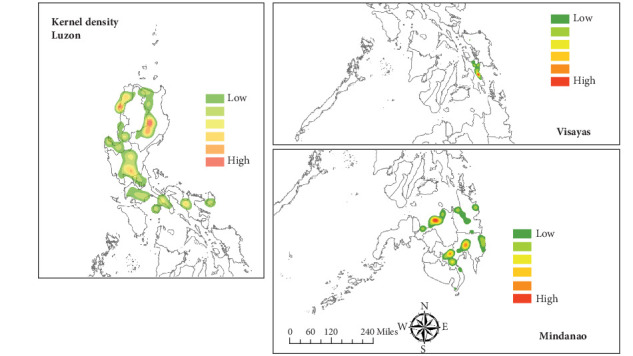
Kernel density estimation.

In terms of outbreak peak timing and progression, data from Luzon most accurately reflect the national epidemic trend, showing a strong and statistically significant correlation with the nationwide outbreak pattern (Spearman's correlation: *ρ* = 0.88, *p* < 0.05; Table 1 and Figure 2), however, pairwise correlations reveal that Luzon's trends differ significantly from those of Visayas and Mindanao (*ρ* = −0.39 and −0.28, respectively; both *p* < 0.001), suggesting distinct regional outbreak patterns across the main islands. KDE (Figure 3) highlighted hotspots and smoothing results in each islands for each local government unit (LGU; in red) based on the 36-month ASF outbreak report. Notably, on Visayas Island, most positive ASF cases are concentrated in Leyte Province, within Region VIII. In contrast, Mindanao exhibits a more widespread distribution of cases, excluding the Autonomous Region in Muslim Mindanao (ARMM).

### 3.2. *R*_*h*_ Estimation

#### 3.2.1. Deterministic Model

**Figure 4:**
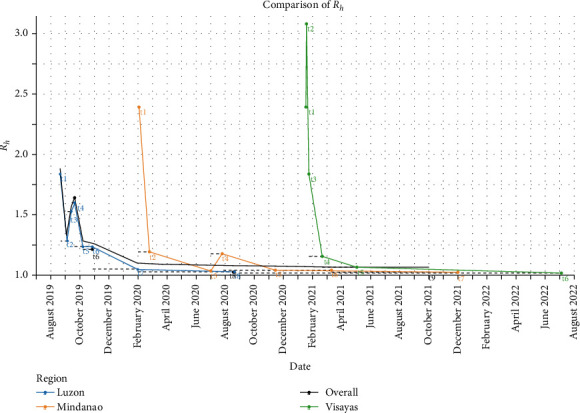
Comparison of the *R*_*h*_ in deterministic model.

In the doubling time method, nine doubling intervals were calculated for the nationwide outbreak, eight for Luzon, seven for Mindanao, and six for Visayas (Table 2 and Figure 4). In the deterministic model, the nationwide estimation showed the highest *R*_*h*_ value of 1.832 during the earliest outbreak in August 2019. Luzon exhibited a similar pattern to the nationwide outbreak. Mindanao recorded its highest *R*_*h*_ value of 2.386 in February 2020 during its initial outbreak, while Visayas showed its peak *R*_*h*_ value of 3.079 in January 2021 during the second doubling time (T2).

#### 3.2.2. Stochastic Model

**Figure 5:**
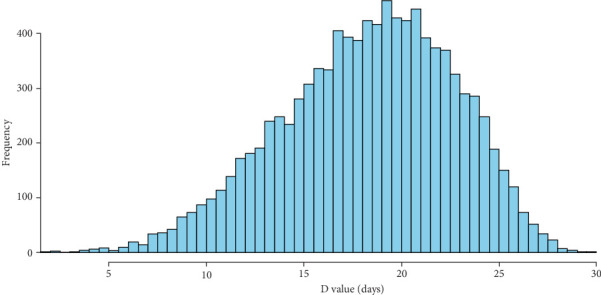
Using a stochastic model to estimate the duration of infectious periods at the farm level.

**Figure 6:**
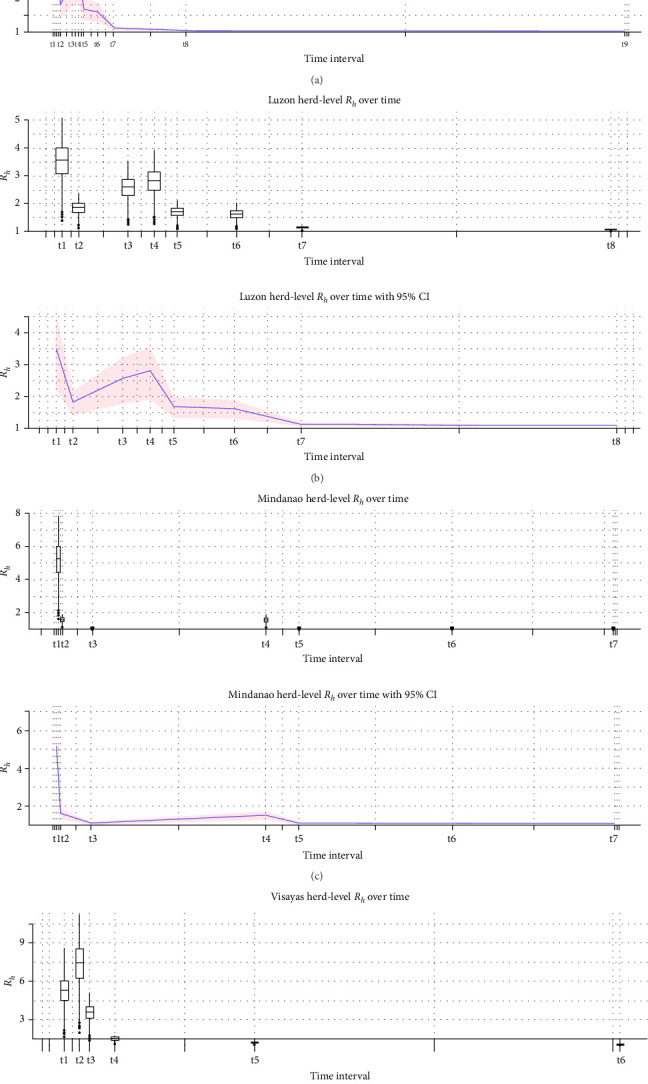
Stochastic model for estimating herd-level *R*_*h*_ over time. (a) National, (b) Luzon, (c) Mindanao, and (d) Visayas.

The modeled infectious period shows the distribution of D values (in days), with the majority clustering around 20 days. The data follow a roughly symmetrical, bell-shaped pattern, highlighting a central tendency near 20 days and fewer occurrences at the extremes (5–30 days; Figure 5). In Luzon, the highest mean *R*_*h*_ value was 3.523 (95% CI: 2.246–4.595) during the first doubling interval (T1). Similarly, Mindanao exhibited its highest mean *R*_*h*_ value of 5.204 (95% CI: 3.076–6.992) in T1. In contrast, Visayas displayed the largest *R*_*h*_ range during the second doubling interval (T2), with a mean value of 7.307 (95% CI: 4.114–9.987). For the last two doubling intervals in each of the islands, all *R*_*h*_ values converged toward 1, indicating a slowing of the outbreak (Figure 6 and Table 3).

#### 3.2.3. TD-R Numbers

**Figure 7:**
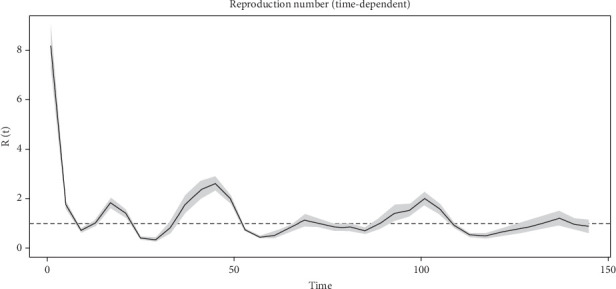
Estimating time-dependent reproductive numbers (TD-R).

The TD-R numbers accounts for weekly potential under-reporting and serves as a complementary approach for characterizing ASF dynamics. At the early phase of ASF outbreak, TD-R values exceeded 8, reflecting high-magnitude initial transmission. Over time, the estimates fluctuated but generally converged toward values oscillating around 1 (Figure 7). This pattern suggests a transition from epidemic spread to endemic transmission at the national scale in the Philippines.

#### 3.2.4. Sensitivity Analysis

Scenario-based sensitivity analyses of both deterministic and stochastic models showed only minor changes in *R*_*h*_ (Supporting Information 2: Figure S1 and Supporting Information 3: Figure S2). Most metrics remained stable on the simulated results, with slight reductions in *T*_*d*_ during the later epidemic phase. These findings indicate that adjusting for plausible levels of under-reporting did not materially alter the estimates of *R*_*h*_ or the epidemic dynamics.

### 3.3. ARIMA Model

**Figure 8:**
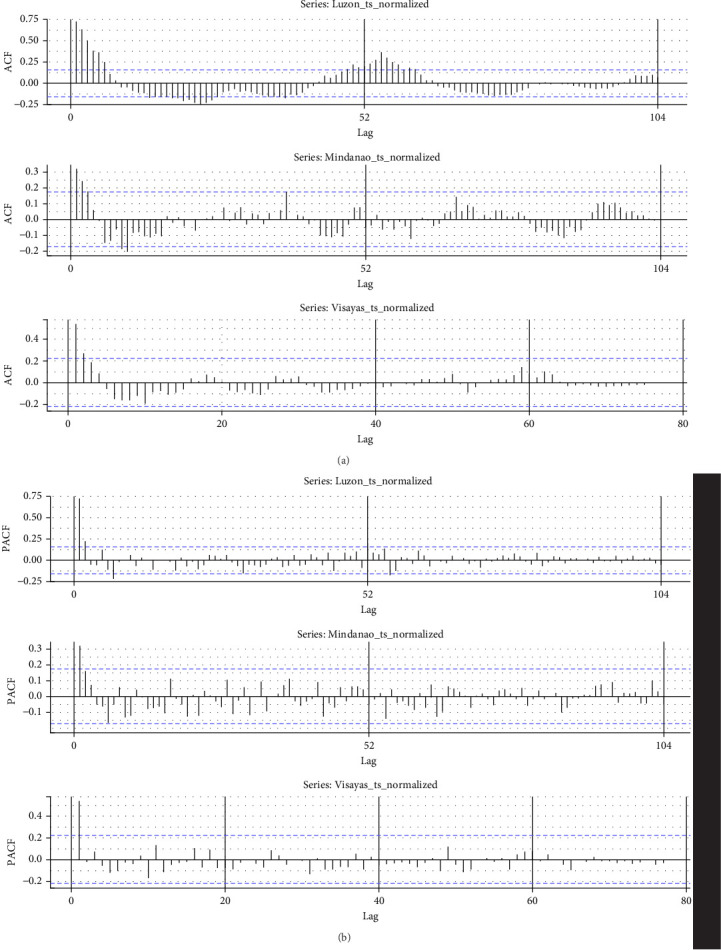
(a) ACF result for each island. (b) PACF result for each island.

The ACF plots (Figure 8) for Luzon, Mindanao, and Visayas suggest potential temporal patterns in ASF outbreak dynamics. Luzon shows strong initial autocorrelation that gradually diminishes at Week 7, with a possible seasonal peak observed around lag weeks 50–60 (notably significant at Week 52), indicating a potential yearly pattern. Mindanao exhibits weaker autocorrelation with some irregular periodicity, implying less consistent temporal trends. Visayas also shows a decline in autocorrelation, with a subtle pattern around lag 52, though it is not statistically significant. Overall, Luzon appears to exhibit the most consistent periodic trend, while Mindanao and Visayas display greater variability.

## 4. Discussion

The first outbreak of ASF occurred on Luzon Island [8]. Because of the distribution of the susceptible population, 79% of the data originates from Luzon, whereas the remaining 21% comes from Mindanao and the Visayas islands. In the estimation of the reproduction number (*R*_*h*_), all methods employed—including deterministic, stochastic doubling time, and TD-R models—indicate that the long-term trend of ASF approaches an *R*_*h*_ value near 1. This suggests a shift towards an endemic pattern of the disease. However, the *R*_*h*_ value does not consistently fall below 1, which implies that while the transmission of ASF has slowed, outbreaks continue to occur. These ongoing outbreaks affect different provinces and LGUs, underscoring the persistent spread of the disease despite control efforts. The potential shift of ASF toward an endemic pattern warrants a thoughtful policy review—particularly in terms of assessing the long-term viability of the total culling strategy. From a sustainability perspective, this evolving situation calls for setting realistic expectations, including the likelihood of periodic fluctuations in outbreak activity. It also underscores the importance of strategic resource allocation for long-term risk communication, ensuring that producers and veterinarians remain well-informed and prepared. Should the current situation persist, it may be appropriate to explore a gradual transition from an eradication-focused framework to a more sustainable disease management approach, with the aim of mitigating the overall impact on the swine sector.

Importantly, the dynamics of ASF transmission appear to differ across the main three islands. Luzon, identified as the initial epicenter of the outbreak and characterized by the highest density of swine farms, exhibits transmission patterns that closely mirror the national trend. Temporal analysis using ARIMA models indicates a recurring yearly pattern in Luzon, with a pronounced increase in outbreak cases typically observed between September and December most years. This seasonal pattern is further supported by a 52-week lag structure identified in the ARIMA results, suggesting a cyclical recurrence of ASF outbreaks in this region during the study period. Conversely, Mindanao and Visayas exhibit distinct transmission dynamics, likely influenced by differences in production systems (e.g., the ratio of commercial farms to backyard farms), demographic structures, and swine population densities. For instance, Luzon's swine industry is characterized by larger, more interconnected commercial farms [23], while Mindanao and Visayas include a higher proportion of backyard farming systems [24, 25], which may alter transmission pathways and control effectiveness. Moreover, the lack of seasonal trends in these regions may reflect their more recent ASF introduction, limiting the time to detect recurring patterns.

Another potential explanation comes from the Philippine Statistics Authority's annual swine report [25]. Before the ASF outbreak (prior to 2019), the swine industry was predominantly centered in Central Luzon (Region III) and Calabarzon (Region IV). However, during the severe 2019–2021 ASF epidemic, the main swine production shifting to regions in the Visayas surpassed Luzon island in terms of swine inventory. This shift resulted in a higher population of susceptible farms in the Visayas. Consequently, when a new epidemic was introduced on Visayas, the *R*_*h*_ value was higher compared to the initial epidemic in Luzon. Alternatively, or additionally, the high *R*_*h*_ value initially observed in Visayas may be consistent with point source infection (or multiple simultaneous introductions) which may have artificially increased the value of the parameter.

Differences in each of the main islands and their corresponding disease dynamic has significant implications for ASF management. A recent study using a semiquantitative risk scoring method identified parts of the Visayas (Iloilo, Aklan, Capiz, and Leyte) as high-risk areas due to high ASF meat contamination levels and a high proportion of backyard farming [26]. The observed variability in disease risk classification suggests that one-size-fits-all management strategy is unlikely to be effective. Island-specific strategies should be considered, taking into account local production systems, geographic spread or zoning status, farm densities, and societal factors. For Luzon, where large-scale commercial farming dominates, strategies may focus on enhancing biosecurity measures and early detection. In contrast, Mindanao and Visayas may benefit from community or village-based surveillance and education programs targeting backyard farmers to curb localized transmission [27, 28].

Recognizing these differences and tailoring ASF control measures accordingly could improve the overall effectiveness of ASF management in the Philippines. Moreover, designing and implementing island-specific strategies not only aligns with the unique transmission patterns observed but also sets realistic expectations for the disease's trajectory on each main islands. This approach could potentially lower the economic impact of ASF and support the long-term recovery of the swine industry across the country.

Studies on the basic reproductive number of ASF in Asian countries remain relatively limited. Currently, research from Vietnam estimates the within-herd *R*_0_ for ASF transmission among fattening pigs and sows in two commercial pig farms to range from 1.08 to 5.38 [29]. Similarly, in South Korea, the *R*_0_ for ASF in wild boars has been estimated to range from 1.11 to 2.37, with a mean value of 1.54 [30]. Further research is needed to understand ASF dynamics in the region.

Our research has limitations. First, the dataset used in this study is based on outbreak centroids provided by the Department of Agriculture. While this spatial resolution is adequate for national- and island-level analysis, it lacks the granularity required for village-level assessment. Second, underreporting and delays in case detection were likely during the early stages of the ASF epidemic in the Philippines, although the extent is difficult to quantify. Some delays in diagnosis and reporting were due to limited diagnostic resources and personnel—challenges also identified in a SWOT analysis of ASF control efforts in the country [31]. As a result, the actual number of outbreaks likely exceeds those captured in the dataset. Therefore, our estimates of *R*_*h*_ may be conservative. We acknowledge that underreporting is an inherent limitation of this study, and we were not able to directly quantify its extent. Although the degree of underreporting likely varied across locations and over time, the proportional changes and relative trends we observed should still provide meaningful insights, even if the absolute number of reported cases was potentially underestimated. In addition, the scenario-based sensitivity analysis (Supporting Information 2: Figure S1 and Supporting Information 3: Figure S2) supports the robustness of our findings regarding the epidemic's dynamics. A further limitation is that surveillance capacity—including the availability of diagnostic kits, laboratory access, and timeliness of detection—varies across regions in the Philippines. An inherent assumption in our analysis is that surveillance sensitivity was consistent across islands and over time. Despite these limitations, ASF is a reportable disease to WOAH, and local government veterinarians routinely collect and report data to the central level. Moreover, the Philippines has a fairly centralized veterinary services and has implemented a nation controlling plan since the beginning of ASF outbreak. For the purpose of this study, we assumed that the reporting bias and diagnostic processes were largely standardized nationwide.

Several unmodeled factors may have influenced the observed transmission patterns. In particular, hog traders, who serve as intermediaries in the pork supply chain, are recognized contributors to human-mediated ASF transmission between islands. Additionally, seasonal environmental variables (temperature and humidity) and restocking practices—such as the Bureau of Animal Industry's recommendation on hog raisers for a minimum 6 month before restocking in ASF-affected areas [32]—while relevant, were not explicitly incorporated due to data limitations, representing further study limitations for temporal trends. The methodological approaches used in this paper assume that outbreaks were caused through transmission from previous outbreaks reported in the same group of islands, and thus, did not account for between-island transmission. This limitation may have resulted in an overestimation of the value of *R*_*h*_ when the indicator was computed separately for each group of islands, because certain cases caused by the re-introduction of ASF from other islands may have been erroneously attributed to within-island transmission. However, because the analyses were not conducted individually for each island in the country, but for three major groups of islands, there is no reason to believe that introductions from neighboring groups of islands are frequent enough to modify the conclusions presented in this paper.

We also considered whether wild boar contributed to transmission in our dataset, using the same study period and cross-validation with EMPRES-i dashboard, a web-based platform developed by FAO's Emergency Prevention System. Only one confirmed wild boar case was reported in Abra Province (Northern Luzon) [33], while the rest—thousands plus of cases—were all in domestic pigs during 2019–2022. Thus, the estimated *R*_*h*_ in our study reflects transmission among domestic pigs only. However, recent evidence from other studies [34–36] has also shown the detection of ASF in both Mindanao and Visayas islands, including PCR detection in Visayan warty pigs [37]. The potential role of wild pigs in ASF spread is a factor that still should be considered, especially given that four known species of Philippine warty pigs (*Sus philippensis*, *S. cebifrons*, *S. oliveri*, and *S. ahoenobarbus*) exist and are threatened by ASF. Although wild boar ASF cases have been reported in multiple local news [38], large-scale systematic surveillance data are currently unavailable. This emphasizes the need for continued target monitoring.

A limitation of the doubling time method is its suitability primarily for evaluating early outbreak dynamics of an infectious disease. The mathematical method assumes exponential growth of cases, which may not always hold true. As the outbreak progresses over the long term, the doubling interval typically increases, potentially introducing inherent bias, particularly when *R*_*h*_ values approach 1. Additionally, estimating the duration of farm infectiousness under different epidemiological scenarios is challenging, making it difficult to account for variations. To address uncertainty and variability, we incorporated a stochastic modeling approach. The TD-R number method was also applied to overcome the doubling time assumption's limitations and assess whether observed trends remain consistent throughout the course of the epidemic.

In conclusion, this study suggests that ASF in the Philippines is shifting toward an endemic status, as indicated by the reproduction number approaching 1 across deterministic, stochastic, and time-dependent models. However, regional transmission dynamics vary significantly from island to island. Luzon island follows the national trend with recurrent outbreaks and seasonal patterns, while Visayas and Mindanao exhibit distinct epidemic trajectories influenced by factors such as swine demographics and production systems. These spatial and temporal differences underscore the need for continuous surveillance and region-specific analyses. A uniform national strategy may be inadequate. Instead, targeted interventions—such as public–private partnerships between swine industry and government in Luzon and community-based surveillance in Visayas and Mindanao—are essential for effective and sustainable long-term ASF control. To address this evolving situation, a shift from epidemic response to long-term endemic management is needed, with strategies tailored to each region's unique context.

## Figures and Tables

**Table 1:** ASF outbreak at national and island level and Spearman correlation.

	ASF outbreak period					Spearman correlation	
	Start date	End date	Total days	Cumulative cases	%	Correlation coefficient (rho)	*p*-Value
Philippines	August 16, 2019	July 20, 2022	1069	19,742	100	—	—
Luzon	August 16, 2019	July 20, 2022	1069	15,526	79	0.8836	<0.001
Mindanao	January 30, 2020	July 15, 2022	897	3482	18	0.0951	0.2406
Visayas	January 12, 2021	July 05, 2022	539	734	4	−0.1337	0.0982

**Spearman pairwise correlation between islands**							

	***ρ* (Spearman)**					***p*-Value**	

Luzon–Visayas	−0.3849					<0.0001	
Luzon–Mindanao	−0.2796					0.0004	
Visayas–Mindanao	0.4355					<0.0001	

**Table 2:** Deterministic model for *R*_*h*_ results.

Doubling timepoint	Start	End	*T*_*d*_ (days)	*R*_*h*_
**Whole country**				

t1	August 16, 2019	August 21, 2019	5	1.832
t2	August 21, 2019	September 05, 2019	15	1.277
t3	September 05, 2019	September 13, 2019	8	1.520
t4	September 13, 2019	September 20, 2019	7	1.594
t5	September 20, 2019	October 8, 2019	18	1.231
t6	October 8, 2019	October 28, 2019	20	1.208
t7	October 28, 2019	January 30, 2020	94	1.044
t8	January 30, 2020	August 17, 2020	200	1.021
t9	August 17, 2020	September 30, 2021	409	1.010
Mean	—	—	—	1.304
Median	—	—	—	1.231

**Luzon**				

t1	August 16, 2019	August 21, 2019	5	1.832
t2	August 21, 2019	September 05, 2019	15	1.277
t3	September 05, 2019	September 13, 2019	8	1.520
t4	September 13, 2019	September 20, 2019	7	1.594
t5	September 20, 2019	October 8, 2019	18	1.231
t6	October 8, 2019	October 28, 2019	20	1.231
t7	October 28, 2019	January 30, 2020	94	1.044
t8	January 30, 2020	August 19, 2020	202	1.021
Mean	—	—	—	1.344
Median	—	—	—	1.254

**Mindanao**				

t1	January 30, 2020	February 02, 2020	3	2.386
t2	February 02, 2020	February 24, 2020	22	1.189
t3	February 24, 2020	June 30, 2020	127	1.033
t4	June 30, 2020	July 24, 2020	24	1.173
t5	July 24, 2020	November 13, 2020	112	1.037
t6	November 13, 2020	March 11, 2021	118	1.035
t7	March 11, 2021	November 29, 2021	263	1.016
Mean	—	—	—	1.267
Median	—	—	—	1.037

**Doubling timepoint**	**Start (first case)**	**End**	***T*_*d*_ (days)**	***R*_*h*_**

**Visayas**				

t1	January 12, 2021	January 15, 2021	3	2.386
t2	January 15, 2021	January 17, 2021	2	3.079
t3	January 17, 2021	January 22, 2021	5	1.832
t4	January 22, 2021	February 19, 2021	28	1.149
t5	February 19, 2021	May 03, 2021	73	1.057
t6	May 03, 2021	July 05, 2022	428	1.010
Mean	—	—	—	1.752
Median	—	—	—	1.490

**Table 3:** Stochasticmodel at national and island level.

Interval	Date	Mean *R*_*h*_	Lower CI (2.5%)	Upper CI (97.5%)
**National result**				

t1	August 16, 2019	3.523	2.246	4.595
t2	August 21, 2019	1.841	1.415	2.198
t3	September 05, 2019	2.577	1.779	3.247
t4	September 13, 2019	2.802	1.890	3.568
t5	September 20, 2019	1.701	1.346	1.999
t6	October 8, 2019	1.631	1.311	1.899
t7	October 28, 2019	1.134	1.066	1.191
t8	January 30, 2020	1.063	1.031	1.090
t9	August 17, 2020	1.031	1.015	1.044

**Luzon**				

t1	August 16, 2019	3.523	2.246	4.595
t2	August 21, 2019	1.841	1.415	2.198
t3	September 05, 2019	2.577	1.779	3.247
t4	September 13, 2019	2.802	1.890	3.568
t5	September 20, 2019	1.701	1.346	1.999
t6	October 8, 2019	1.631	1.311	1.899
t7	October 28, 2019	1.134	1.066	1.191
t8	January 30, 2020	1.062	1.031	1.089

**Mindanao**				

t1	January 30, 2020	5.204	3.076	6.992
t2	February 02, 2020	1.573	1.283	1.817
t3	February 24, 2020	1.099	1.049	1.142
t4	June 30, 2020	1.526	1.260	1.749
t5	July 24, 2020	1.113	1.056	1.160
t6	November 13, 2020	1.107	1.053	1.152
t7	March 11, 2021	1.048	1.024	1.068

**Visayas**				

t1	January 12, 2021	5.204	3.076	6.992
t2	January 15, 2021	7.307	4.114	9.987
t3	January 17, 2021	3.523	2.246	4.595
t4	January 22, 2021	1.450	1.222	1.642
t5	February 19, 2021	1.173	1.085	1.246
t6	May 03, 2021	1.029	1.015	1.042

## Data Availability

The data are the property of the Philippine Government and have been shared with us to conduct the research supporting training activities in the country. Any requests for access should be directed to the Philippine Bureau of Animal Industry and to the International Training Center on Pig Husbandry of the Philippines.

## Supporting Information

Supporting Information 1 The deterministic model results for calculating the reproduction number (*R*_*h*_) across the full range of infectious periods are provided.

Supporting Information 2 Figure S1: National-level simulation.

Supporting Information 3 Figure S2: Island-level simulation.
