# Supplementary figures and images for: Estimation of the Farm-Level Basic Reproduction Number for African Swine Fever Outbreaks in the Philippines
Source: Transbound Emerg Dis. 2025 Oct 23;2025:6946683. doi: 10.1155/tbed/6946683 (PMC12575035; doi:10.1155/tbed/6946683)

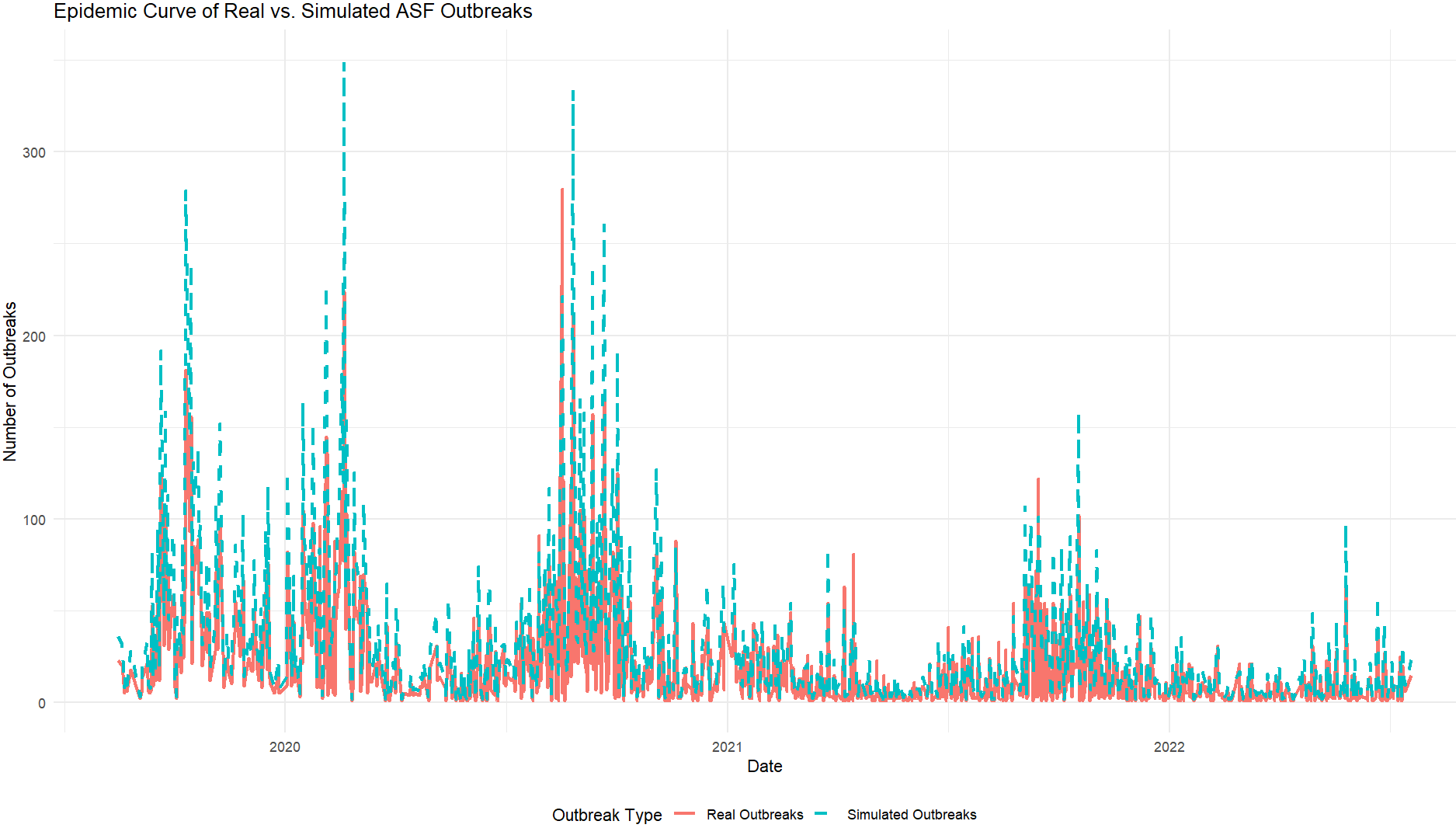

Supplement: Supporting Information 2 — Figure S1: National-level simulation. [file 6946683.f2.png]

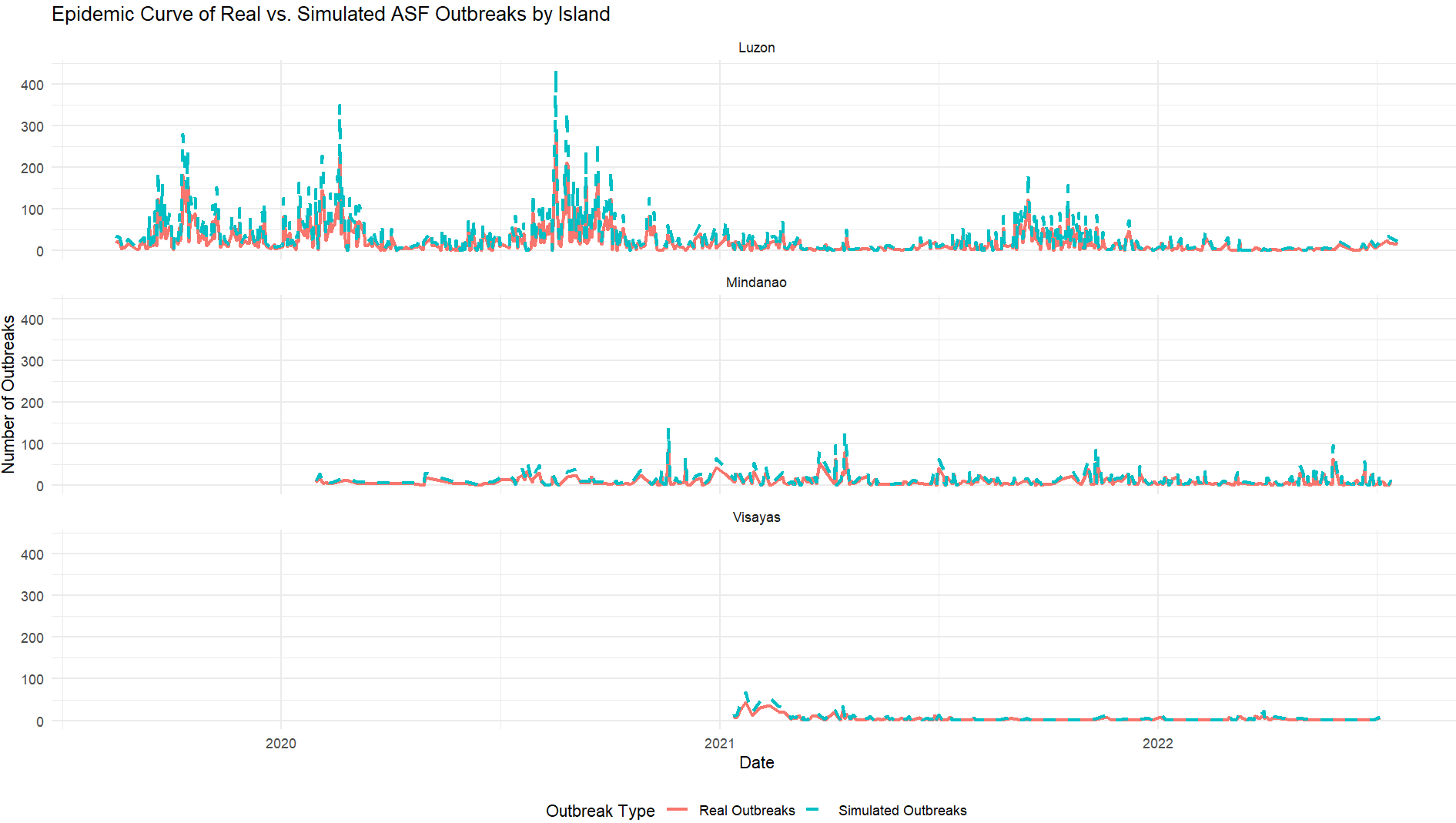

Supplement: Supporting Information 3 — Figure S2: Island-level simulation. [file 6946683.f3.png]
